# Exploring the Diversity and Antimicrobial Potential of Marine Actinobacteria from the Comau Fjord in Northern Patagonia, Chile

**DOI:** 10.3389/fmicb.2016.01135

**Published:** 2016-07-19

**Authors:** Agustina Undabarrena, Fabrizio Beltrametti, Fernanda P. Claverías, Myriam González, Edward R. B. Moore, Michael Seeger, Beatriz Cámara

**Affiliations:** ^1^Laboratorio de Microbiología Molecular y Biotecnología Ambiental, Departamento de Química & Centro de Biotecnología Daniel Alkalay Lowitt, Universidad Técnica Federico Santa MaríaValparaíso, Chile; ^2^Actygea S.r.l.Gerenzano, Italy; ^3^Culture Collection University of Gothenburg (CCUG), Sahlgrenska Academy, University of GothenburgGothenburg, Sweden; ^4^Department of Infectious Diseases, Sahlgrenska Academy, University of GothenburgGothenburg, Sweden

**Keywords:** cultivable actinobacteria, antimicrobial activity, Comau fjord, marine sediments, Northern Patagonia

## Abstract

Bioprospecting natural products in marine bacteria from fjord environments are attractive due to their unique geographical features. Although, *Actinobacteria* are well known for producing a myriad of bioactive compounds, investigations regarding fjord-derived marine *Actinobacteria* are scarce. In this study, the diversity and biotechnological potential of *Actinobacteria* isolated from marine sediments within the Comau fjord, in Northern Chilean Patagonia, were assessed by culture-based approaches. The 16S rRNA gene sequences revealed that members phylogenetically related to the *Micrococcaceae, Dermabacteraceae, Brevibacteriaceae, Corynebacteriaceae, Microbacteriaceae, Dietziaceae, Nocardiaceae*, and *Streptomycetaceae* families were present at the Comau fjord. A high diversity of cultivable *Actinobacteria* (10 genera) was retrieved by using only five different isolation media. Four isolates belonging to *Arthrobacter, Brevibacterium, Corynebacterium* and *Kocuria* genera showed 16S rRNA gene identity <98.7% suggesting that they are novel species. Physiological features such as salt tolerance, artificial sea water requirement, growth temperature, pigmentation and antimicrobial activity were evaluated. *Arthrobacter, Brachybacterium, Curtobacterium, Rhodococcus*, and *Streptomyces* isolates showed strong inhibition against both Gram-negative *Pseudomonas aeruginosa, Escherichia coli* and *Salmonella enterica* and Gram-positive *Staphylococcus aureus, Listeria monocytogenes*. Antimicrobial activities in *Brachybacterium, Curtobacterium*, and *Rhodococcus* have been scarcely reported, suggesting that non-mycelial strains are a suitable source of bioactive compounds. In addition, all strains bear at least one of the biosynthetic genes coding for NRPS (91%), PKS I (18%), and PKS II (73%). Our results indicate that the Comau fjord is a promising source of novel *Actinobacteria* with biotechnological potential for producing biologically active compounds.

## Introduction

The increased prevalence of multi-drug resistance pathogens along with the rapid development of cross resistances with new antibiotics is the driving force in the identification and production of novel therapeutic agents (Livermore, 2009). All classes of antibiotics have seen emergence of resistance compromising their use; hence there is an urgent need for new bioactive compounds (Genilloud, 2014). The traditional approach consisting of isolation and cultivation of new microorganisms of underexplored habitats is still rewarding (Axenov-Gribanov et al., 2016), and has brought to the identification, production and commercialization of most of the antibiotics (Newman and Cragg, 2012). Despite the chemically synthetic efforts, natural environments are still the main source for the discovery of novel antibiotics (Fenical and Jensen, 2006; Bull and Stach, 2007). Although, the diversity of life in terrestrial environments is well reported, the highest biodiversity is in the world's oceans (Donia and Hamann, 2003). Oceans are strongly complex habitats in terms of pressure, salinity and temperature variations (Fenical, 1993), therefore marine microorganisms have to develop physiological traits including chemically complex biosynthesized metabolites to ensure their survival in this highly dynamic habitat. Research has taken advantage from these unique molecules to discover novel bioactive compounds with antibacterial, antifungal and/or antitumor properties, and apply them in current clinical challenges (Gulder and Moore, 2010).

In this scenario, bacteria from the *phylum Actinobacteria* are a prominent source of biologically active natural compounds, since they are well known for their capacity to biosynthesize versatile secondary metabolites (Katz and Baltz, 2016). Actinobacteria are one of the major phyla of the domain *Bacteria* (Goodfellow and Fiedler, 2010). It encompasses high GC-content Gram-positive bacteria that includes 17 orders (Gao and Gupta, 2005; Sen et al., 2014). Surprisingly, the class *Actinobacteria* contains both the most deadly bacterial pathogen (i.e., *Mycobacterium* genus) and the microorganisms that are the most important for antibiotic production (i.e., *Streptomyces* genus) (Doroghazi and Metcalf, 2013). *Streptomyces* are responsible for two-thirds of all known antibiotics. In addition, several other important biologically-active compounds have been found, including antitumoral, antifungal, herbicidal, and antiparasitic compounds (Bérdy, 2005). Due to the extensive sampling of soil *Streptomyces*, the rate of discovery of novel metabolites is decreasing (Fenical, 1993), which is the reason why bioprospecting efforts are currently being developed in marine underexplored ecosystems.

Marine environments are an established ecological niche for actinobacteria (Das et al., 2006; Ward and Bora, 2006). Cultivable actinobacteria from marine habitats have been characterized from mangrove forests (Hong et al., 2009; Baskaran et al., 2011; Lee et al., 2014a,b; Ser et al., 2015, 2016), marine sponges (Kim et al., 2005; Montalvo et al., 2005; Zhang et al., 2006; Jiang et al., 2007; Sun et al., 2015), corals (Hodges et al., 2012; Kuang et al., 2015; Mahmoud and Kalendar, 2016; Pham et al., 2016), sea cucumbers (Kurahashi et al., 2010), pufferfishes (Wu et al., 2005), and seaweed (Lee et al., 2008). Notably, actinobacteria are predominant in marine sediments (Mincer et al., 2002; Magarvey et al., 2004; Jensen et al., 2005; Bredholdt et al., 2007; Gontang et al., 2007; León et al., 2007; Maldonado et al., 2008; Duncan et al., 2014; Yuan et al., 2014) and also in deep sea sediments (Colquhoun et al., 1998; Pathom-Aree et al., 2006). Marine actinobacteria have been described as an emerging source for novel bioactive molecules (Lam, 2006; Joint et al., 2010; Subramani and Aalbersberg, 2012; Zotchev, 2012). The majority of these secondary metabolites are produced by polyketide synthases (PKS) and non-ribosomal peptide synthetases (NRPS) metabolic pathways (Salomon et al., 2004). Notably, it is reported that actinobacteria have a higher number of these biosynthetic genes (Donadio et al., 2007).

The extensive coast of Chile is a promising biome to explore marine actinobacterial communities, and in this context, the bioprospecting of sediments of a marine protected area, the Comau fjord, in the Chilean Northern Patagonia was proposed. The Comau fjord is a pristine area unique by its geological nature. It is comparatively smaller than other fjords in Chile, and also one of the deepest (Ugalde et al., 2013); characterized by steep slopes, with surrounding mountains that have a height of up to 2000 m with a dense extratropical rainforest covering from the sea to the top (Lagger et al., 2009). The aim of this study was to isolate marine actinobacteria from this unique ecosystem. The cultivable diversity of actinobacterial strains along with their environmental adaptation traits was investigated, and their ability to produce antibacterial activity against model strains was assessed.

## Materials and methods

### Environmental samples

**Figure 1:**
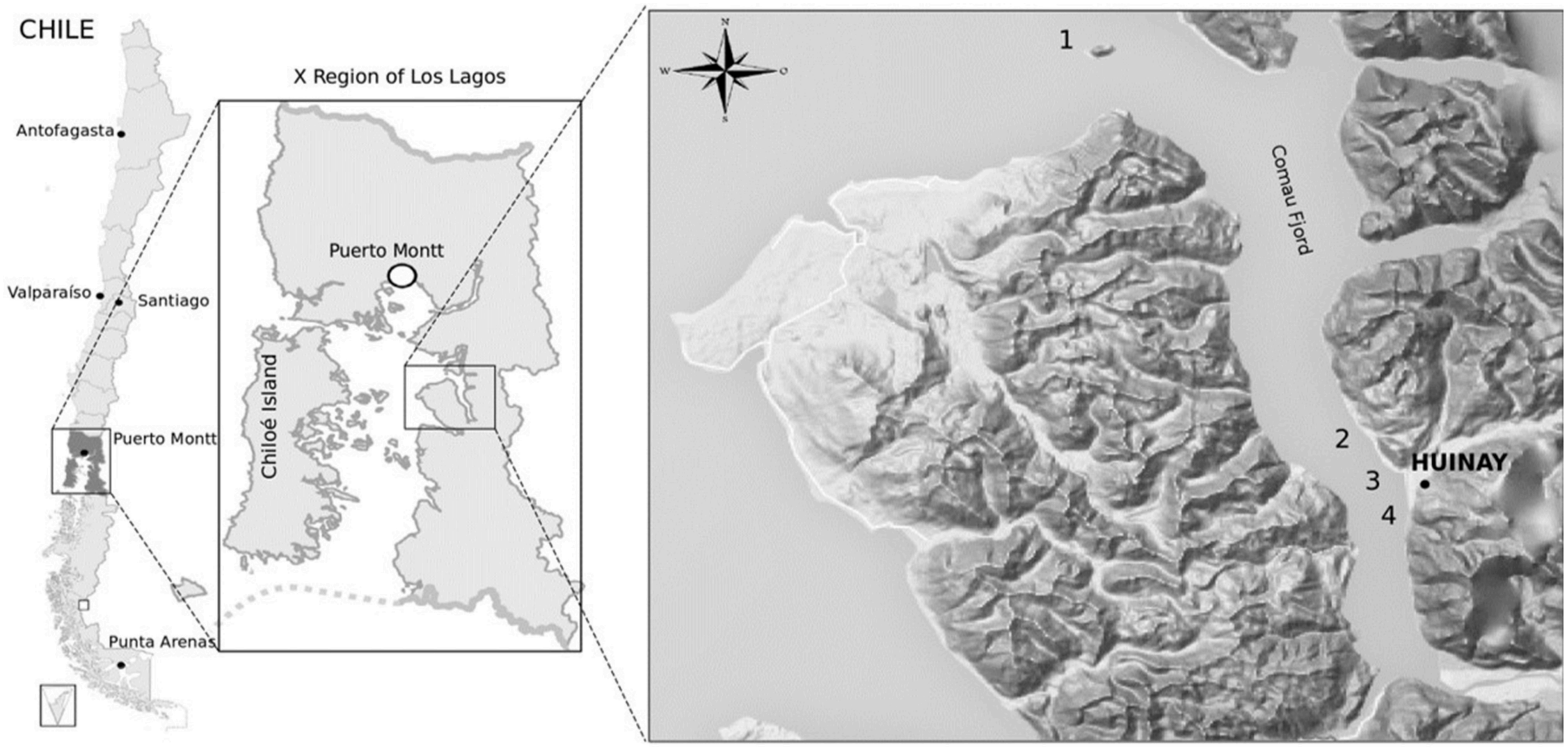
**Geography of sampling sites for actinobacteria isolation from the Comau fjord in Northern Patagonia, Chile**. Map of sampling locations within the Comau fjord (Los Lagos Region). Numbers indicate the sites where marine sediments were collected at the coast close to: Lilihuapi Island (1), Punta Llonco (2), Lloncochaigua River mouth (3), and Tambor Waterfall (4). Black dot indicates location of the Huinay Scientific Field Station.

Sampling was performed in the Marine Protected Area of Huinay in January 2013, located in the Commune of Hualaihué, in the Los Lagos Region, Chile. Samples were collected from marine sediments within the Comau Fjord in the Northern Patagonia. Four different coastal locations were sampled in front of Lilihuapi Island (42°20, 634′S; 72°27, 429′W), Tambor Waterfall (42°24, 161′S; 72°25, 235′W), Punta Llonco (42°22, 32′S; 72°25, 4′W), and in front of Lloncochaigua River mouth (42°22, 37′S; 72°27, 25′W) (Figure 1). Underwater samples were collected by Huinay Scientific Field Station scuba divers, dispensing samples directly from marine sediments into sterile 50 mL tubes. Marine sediments were taken from subtidal zones at different depths, ranging from 0.25 to 26.2 m. Salinity was measured at each sampling site, and ranged from 5 μg L^−1^ in the coast in front of Lloncochaigua River mouth, where there is a meaningful input of fresh water, to 31 μg L^−1^ in the coast of Lilihuapi Island, located further away from continental land. Samples were maintained on ice until transported to the laboratory, where they were stored at 4°C.

### Isolation of actinobacteria

Samples were both plated directly or serially diluted (10^−4^ and 10^−6^) before plating on selective media for the isolation of actinobacteria. Five selective media were used as previously reported (Claverías et al., 2015): M1 Agar (Mincer et al., 2002), ISP2 and NaST21Cx Agar (Magarvey et al., 2004), R2A Agar (Difco), and Marine Agar (MA) 2216 (Difco). All media were amended with nalidixic acid (25 μg mL^−1^), as an inhibitor of primarily fast-growing Gram-negative bacteria, and cycloheximide (100 μg mL^−1^) for fungi inhibition [28]. All media with the exception of Marine Agar, were prepared with artificial sea water (ASW) (Kester et al., 1967). The agar media cultures were incubated at 30°C until visible colonies were observed, up to 1–2 months. For isolation purposes, colonies were individually streaked out onto Tryptic Soy Agar medium (TSA) prepared with ASW (TSA-ASW) and eventually transferred on new plates until pure cultures were obtained. Isolated bacteria were stored at −20 and −80°C, in 20% glycerol, TSB medium and ASW for maintenance.

### Detection and identification of actinobacteria

A PCR-assay was conducted as a screening method for detecting actinobacterial strains among the isolates with primers targeting the V3–V5 regions of the 16S rRNA gene of actinobacteria (S-C-Act-0235-a-S-20 and S-C-Act-0878-A-19) (Stach et al., 2003). DNA extractions were performed, using a lysis method by culture boiling suspensions of bacterial cells (Moore et al., 2004). Each PCR reaction contained 1 μL of genomic DNA, 12.5 μL of GoTaq Green Master Mix (Promega) and 0.6 μM of each primer in a final reaction volume of 25 μL. The reaction started with an initial denaturation, at 95°C for 5 min, followed by 35 cycles of DNA denaturation, at 95°C for 1 min, primer-annealing, at 70°C for 1 min and extension cycle, at 72°C for 1.5 min, with a final extension at 72°C for 10 min (Claverías et al., 2015). PCR-amplicons were visualized in 2% agarose gel electrophoresis and subsequently revealed with SYBR Green staining (E-gel, Invitrogen).

Positive isolates were selected for 16S rRNA gene amplification, using universal primers 27F and 1492R (Lane, 1991). The reaction mix (50 μL) contained 1 μL of genomic DNA, 25 μL of GoTaq Green Master Mix (Promega) and 0.2 μM of each primer. The reaction started with an initial DNA denaturation at 95°C for 5 min, followed by 30 cycles of denaturation at 95°C for 1 min, primer-annealing at 55°C for 1 min and primer-extension at 72°C for 1.5 min, with a final extension at 72°C for 10 min. PCR products were sent to Macrogen Inc. (Seoul, Korea) for purification and sequencing using the conserved universal primer 800R. Retrieved sequences were manually edited and BLAST nucleotide analyses were performed with the National Center for Biotechnology Information server (NCBI) and actinobacteria were initially identified up to the genus level.

### Antimicrobial activity tests

Bioprospecting for antimicrobial activity was initially performed using the cross-streak method as described (Haber and Ilan, 2014), with slight modifications (Claverías et al., 2015). Fresh cultures of the isolated actinobacterial strains were inoculated as a line in the middle of an agar medium plate and incubated at 30°C until notable growth was observed (7 days for mycelial strains and 5 days for non-mycelial strains). Strains were grown on TSA-ASW and ISP2-ASW media. Five reference bacteria were the target of inhibition tests: *Staphylococcus aureus* NBRC 100910^T^ (STAU); *Listeria monocytogenes* 07PF0776 (LIMO); *Salmonella enterica* subsp enterica LT2^T^ (SAEN); *Escherichia coli* FAP1 (ESCO) and *Pseudomonas aeruginosa* DSM50071^T^ (PSAU). Cultures were incubated at 37°C overnight and inhibition zones were ranked qualitatively as: −, no inhibition; +/−, attenuated growth of test strain in the area closest to the actinobacterial line; +, <50% growth inhibition (less than half of the bacterial line was inhibited); ++, 50% growth inhibition (half of the bacterial line was inhibited); +++, >50% growth inhibition (more than half of the bacterial line was inhibited). All experiments were performed in duplicate, using an internal control with one of the reference strains.

Further antimicrobial tests were performed with selected isolates *Streptomyces* sp. H-KF8, *Arthrobacter* sp. H-JH3, *Brevibacterium* sp. H-BE7, *Kocuria* sp. H-KB5 and *Rhodococcus* sp. H-CA8f. Strains were grown in a 50 mL liquid culture in ISP2-ASW medium for 10 days for non-mycelial strains and 15 days for the mycelial strain, with continuous shaking at 30°C. Crude extracts were obtained after solvent extraction using hexane, methanol and ethyl acetate in a 1:1 ratio (v/v) for two times. Evaporation of solvent was performed with speed vacuum, and extract was dissolved in 10% dimethyl sulphoxide (DMSO) until a final concentration of 5 mg mL^−1^. Antimicrobial assays were evaluated using 10 μL of each extract, over LB agar plates spread with the bacterial test strains STAU, PSAU, SAEN, and ESCO. Plates were incubated overnight at 37°C and inhibitions zones were checked. ISP2 medium and 10% DMSO were used as negative controls.

### Detection of PKS and NRPS biosynthetic genes

Amplification of biosynthetic genes was carried out by PCR, using degenerate primers targeting the ketosynthase domain in PKS type I with primers KS-F (5′CCSCAGSAGCGCSTS YTSCTSGA3′) and KS-R (5′GTSCCSGTSCCGTGSGYS TCSA3′) (Gontang et al., 2010); and PKS type II with primers KSα (5′TSGRCTACRTCA ACGGSCACGG3′) and KSβ (5′TACSAGTCS WTC GCCTGGTTC3′) (Ayuso et al., 2005). The adenylation domain in NRPS systems was detected with primers A3F (5′GCSTACSYSATSTAC ACSTCSGG3′) and A7R (5′SASGTCVCCSGTS CGGTAS3′) (Ayuso-Sacido and Genilloud, 2005). PCR programs were performed as previously described (Ayuso et al., 2005; Ayuso-Sacido and Genilloud, 2005; Gontang et al., 2010). Products were visualized in 1% agarose gels electrophoresis, and stained with GelRed (Biotium). *Streptomyces violeaceoruber* DSM 40783 was used as a control for all PCR reactions. Detection was determined as +, if the amplicon was located at the expected size (700 bp for PKS type I; 800–900 bp for PKS type II and 700–800 bp for NRPS); and −, if amplicon was absent or it was present at any other size.

### Phylogenetic analysis

Representative strains for each genus identified from partial 16S rRNA gene sequence analyses were selected for the nearly-complete sequencing of this gene, as previously described (Claverías et al., 2015). PCR products were quantified and sent to Macrogen Inc. (Seoul, Korea) for purification and sequencing, using primers 27F, 518F, 800R, and 1492R. Manual sequence edition, alignment, and contig assembling were performed using Vector NTI v10 software package (Invitrogen). Sequence contigs were analyzed performing BLAST with NCBI to determine the closest type strain match using the 16S ribosomal RNA sequence of Bacteria and Archaea database. The Neighbor-Joining algorithm (Saitou and Nei, 1987) using MEGA software version 6.0 (Tamura et al., 2013) with bootstrap values based on 1000 replications (Felsenstein, 1985) was used to construct a phylogenetic tree based on the V1-V9 region of the 16S rRNA gene sequences. The 16S rRNA gene sequences were deposited in GenBank under the following accession numbers: *Arthrobacter* sp. H-JH3 (KT799841); *Brachybacterium* sp. H-CG1 (KT799842); *Brevibacterium* sp. H-BE7 (KT799843); *Corynebacterium* sp. H-EH3 (KT799844); *Curtobacterium* sp. H-ED12 (KT799845); *Kocuria* sp. H-KB5 (KT799846); *Dietzia* sp. H-KA4 (KT799847); *Micrococcus* sp. H-CD9b (KT799848); *Rhodococcus* sp. H-CA8f (KT799849); *Streptomyces* sp. H-KF8 (KT799850) and *Streptomyces* sp. H-CB3 (KT799851).

### Phenotypic characterization of actinobacterial strains

For the morphological and physiological characterization of the representative strains, colony pigmentation, spore formation, growth temperatures, ASW requirement and NaCl tolerance were evaluated. Optimal colony pigmentation was observed on TSA-ASW after a 3-month incubation at 4°C. To establish the effects of temperature on growth, 10 μL of actinobacterial cultures were streaked onto TSA-ASW plates, and incubated at 4, 20, 30, 37, and 45°C. For NaCl tolerance, LB agar with 0, 1, 3.5, 5.0, 7.0, 10, and 20% (w/v) NaCl was prepared. As described previously, 10 μL of the actinobacterial cultures were streaked onto LB agar plates and incubated at 30°C. To detect the requirement of seawater on growth, ISP2 was prepared as follows: medium with Milli-Q H_2_O; medium with ASW; and medium with Milli-Q H_2_O supplemented with 3.5% (w/v) NaCl (equivalent to ASW NaCl concentration). Incubation times were from 10 days (for non-mycelial strains) to 14 days (for mycelial stains) at 30°C. The reference time for growth was that on which growth was observed on control plates. Results were interpreted as: +, if the strain tested was able to grow on medium-ASW but did not grow on medium/Milli-Q H_2_O and on medium/Milli-Q H_2_O supplemented with 3.5% NaCl; and −, if the strain tested was able to grow on all three media.

### Resistance to model antibiotics

Representative strains of each genus were grown to exponential phase (turbidity at 600 nm of 0.3) and plated on Mueller-Hinton agar plates for antibiotic susceptibility testing. Antibiotic discs for Gram-positive bacteria (Valtek) were placed above and inhibition grown zones as diameters were measured and compared with values obtained from the Clinical and Laboratory Standards Institute (CLSI) from year 2016 to determine susceptibility (S), or resistance (R) of each antibiotic tested.

## Results

### Isolation and identification of actinobacteria

**Figure 2:**
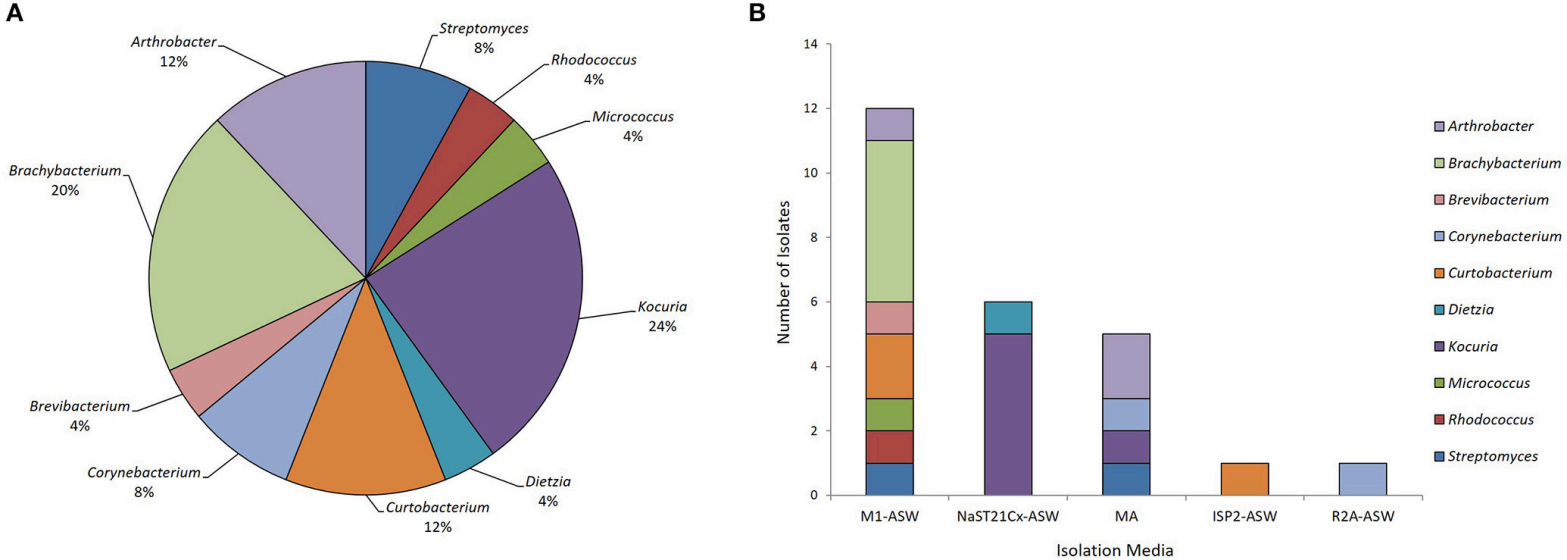
**Biodiversity of actinobacteria from the Comau fjord in Northern Patagonia. (A)** Distribution of the relative abundance of the actinobacterial genera isolated. **(B)** Number of actinobacteria of various genera isolated using different culture media.

Eleven marine sediment samples were collected from four different sites in Comau fjord, Northern Patagonia, Chile (Figure 1). Altogether 25 marine actinobacteria were isolated. Their distribution according to the sampling site was: 40% from Lilihuapi island coast, 28% from Punta Llonco, and 16% from Loncohaigua river mouth and Tambor waterfall, each. The majority (80%) of the isolates were from sediments situated approximately 10 m deep. Only occasional isolates were obtained from deeper sediments or from the shallow locations. The *Actinobacteria* isolated belong to three suborders: *Streptomycineae, Micrococcineae*, and *Corynebcaterineae*; comprising eight different families. Relative abundances of the strains according to the genera isolated (Figure 2A) indicated that most abundant genera were *Kocuria* and *Brachybacterium*. The selective media had a major influence on the number of isolates obtained (Figure 2B). M1-ASW medium was the most effective regarding the number and diversity of isolates recovered. Interestingly, strains of *Brachybacterium, Brevibacterium, Micrococcus*, and *Rhodococcus* genera were isolated exclusively with this medium (Figure 2B).

### Antimicrobial activity assays

**Figure 3:**
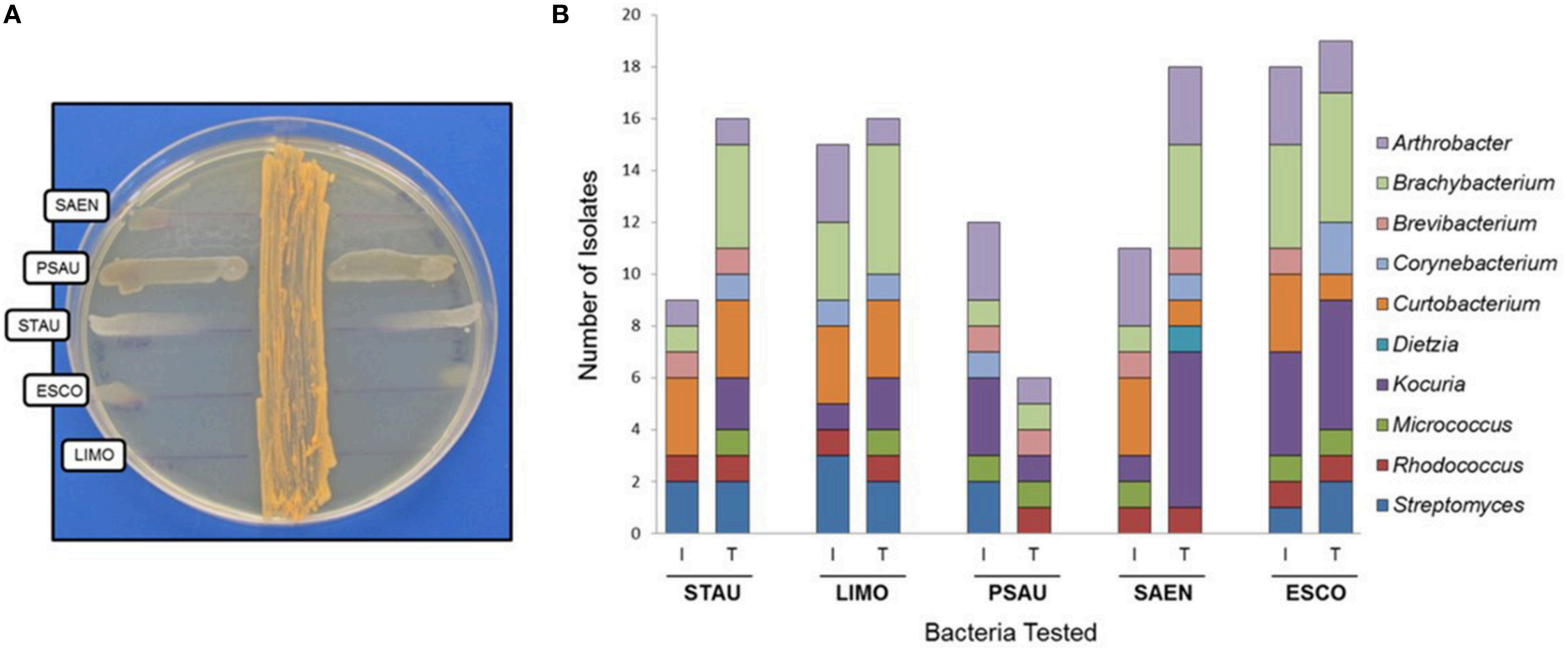
**Antimicrobial activity of actinobacterial strains from the Comau fjord in Northern Patagonia. (A)** Cross-streak method of *Rhodococcus* sp. H-CA8f showing different patterns of inhibition zones with several model bacteria. **(B)** Antimicrobial activity of actinobacterial strains using the cross-streak method. STAU, *Staphylococcus aureus*; LIMO, *Listeria monocytogenes*; PSAU, *Pseudomonas aeruginosa*; SAEN, *Salmonella enterica*; ESCO, *Escherichia coli*. I, ISP2-ASW media; T, TSA-ASW media.

Our first approach was to screen all actinobacterial strains for antimicrobial activity, using the cross-streak method, against five reference strains: STAU, LIMO, PSAU, SAEN, and ESCO (Figure 3A). Actinobacterial strains showed antimicrobial activity, presenting a broad spectrum of inhibition although with different inhibition patterns (Table 1). Inhibition of reference strains largely depended on the media where actinobacterial strains were cultivated, proving TSA-ASW to be generally better for antimicrobial activity than ISP2-ASW medium. *Arthrobacter, Brachybacterium, Curtobacterium*, and *Rhodococcus* isolates showed potent antimicrobial bioactivity to more than one target (Table 1). Regarding the Gram-negative bacteria tested, TSA-ASW-grown actinobacterial strains were able to inhibit ESCO (84%) and PSAU (24%); whereas ISP2-ASW-grown isolates inhibited up to 76 and 48%, respectively. Concerning the Gram-positive reference strains, 64% of the TSA-ASW-grown actinobacterial strains inhibited both LIMO and STAU; whereas ISP2-ASW-grown strains, 56% showed inhibition for LIMO and 36% for STAU (Figure 3B).

**Table 1:** **Antimicrobial activity of actinobacterial strains against model pathogens using the cross-streak method**.

**Strain**	**Genus**	**STAU**		**LIMO**		**PSAU**		**SAEN**		**ESCO**	
		**ISP2**	**TSA**	**ISP2**	**TSA**	**ISP2**	**TSA**	**ISP2**	**TSA**	**ISP2**	**TSA**
H-CA8b	*Arthrobacter*	+/−	+/−	+++	+++	+++	+++	+/−	+/−	++	++
H-JH1	*Arthrobacter*	−	−	++	−	+	−	+/−	+/−	+	−
H-JH3	*Arthrobacter*	−	−	++	−	+	−	+/−	+/−	+	+/−
H-CA4	*Brachybacterium*	−	−	+++	+	−	++	−	+/−	+/−	+++
H-CD1	*Brachybacterium*	−	+/−	−	+++	−	−	−	+/−	+/−	+/−
H-CE9	*Brachybacterium*	−	+/−	+	++	−	−	+	+	++	+
H-CF1	*Brachybacterium*	++	++	+	++	−	−	−	+/−	−	+/−
H-CG1	*Brachybacterium*	−	+/−	−	+++	+/−	−	−	−	+/−	+/−
H-BE7	*Brevibacterium*	+	+/−	−	−	+	+/−	++	+	+	−
H-EH3	*Corynebacterium*	−	+/−	+/−	+++	−	−	−	−	−	+/−
H-KF5	*Corynebacterium*	−	−	−	−	+/−	−	−	+/−	−	+/−
H-BE10	*Curtobacterium*	+++	+++	+/−	+++	−	−	++	+++	+/−	++
H-CD9a	*Curtobacterium*	+	+	+/−	+	−	−	+/−	−	+/−	+/−
H-ED12	*Curtobacterium*	++	++	+	++	−	−	+/−	−	+/−	+/−
H-KA4	*Dietzia*	−	−	−	−	−	−	−	+/−	−	−
H-KA9	*Kocuria*	−	−	−	−	+/−	−	−	+/−	−	+/−
H-KA10	*Kocuria*	−	+/−	−	+/−	−	−	−	+++	+/−	+/−
H-KB1	*Kocuria*	−	−	−	−	−	−	−	+/−	+/−	+/−
H-KB5	*Kocuria*	−	−	−	−	+/−	−	−	+/−	+/−	−
H-KB6	*Kocuria*	−	+/−	+/−	+++	+/−	+/−	+	+/−	+/−	+
H-JH7	*Kocuria*	−	−	−	−	−	−	−	+	−	+/−
H-CD9b	*Microccocus*	−	+/−	−	+++	+	+/−	+/−	−	+/−	+/−
H-CA8f	*Rhodococcus*	++	++	+++	+++	−	+++	+++	+++	+++	+++
H-CB3	*Streptomyces*	+++	+++	+/−	+/−	+/−	−	−	−	+	++
H-KF8	*Streptomyces*	+++	+++	+/−	+/−	+/−	−	−	−	+	+

−, no inhibition; +/−, attenuated growth; +, <50% growth inhibition; ++, 50% growth inhibition; +++, >50% growth inhibition. Both media were prepared with ASW.

Notably, 67% of the antimicrobial activities observed with the cross-streak method were retrieved with various solvent extractions from actinobacterial liquid cultures (Table 2). Ethyl acetate was more effective in extracting active compounds, as crude extracts from *Rhodococcus* sp. H-CA8f, *Kocuria* sp. H-KB5 and *Brevibacterium* sp. H-BE7 presented antimicrobial activity. On the other hand, antimicrobial activity from *Arthrobacter* sp. H-JH3 was effectively extracted from the cell pellet using methanol. Crude extracts from *Rhodococcus* sp. H-CA8f showed an antimicrobial effect against all bacteria tested, confirming results obtained from the cross-streak method.

**Table 2:** **Antimicrobial activities of crude extracts using various solvents for selected actinobacterial isolates grown in ISP2-ASW medium**.

**Strain**	**Solvent**	**Bacterial Test Strain**			
		**STAU**	**PSAU**	**SAEN**	**ESCO**
H-KF8	Hexane	−	−	−	−
	Ethyl acetate	−	−	−	−
	Methanol	+	−	−	+
H-CA8f	Hexane	−	−	−	−
	Ethyl acetate	+	+	+	+
	Methanol	−	−	−	−
H-KB5	Hexane	−	−	−	−
	Ethyl acetate	−	+	+	+
	Methanol	−	−	−	−
H-JH3	Hexane	−	−	−	−
	Ethyl acetate	−	−	−	−
	Methanol	−	−	+	+
H-BE7	Hexane	−	−	−	−
	Ethyl acetate	−	−	+	−
	Methanol	−	−	−	−

### Detection of PKS and NRPS biosynthetic genes

The presence of biosynthetic PKS (type I and II) and NRPS genes were detected by PCR in representative actinobacterial isolates (Table 3). Interestingly, most isolates bear at least one biosynthetic gene of PKS or NRPS. Among them, NRPS was the predominant gene observed (91%), followed by PKS type II (73%). Only 18% of actinobacterial isolates showed the presence of PKS type I gene.

**Table 3:** **Biogeographic and physiological characteristics of representative actinobacterial strains**.

**Strain**	**Closest Type Strain (Accession N°) (% Identity)**	**Biogeographic Characteristics**				**Physiological Characteristics**				**Biosynthetic genes**		
		**Sampling Site**	**Depth (m)**	**Salinity (ppt)**	**Sediment characteristics**	**Temperature (°C)**	**Salinity (%NaCl)**	**ASW Requirement**	**Pigmentation**	**PKS I**	**PKS II**	**NRPS**
H-JH3	*Arthrobacter oxydans* DSM 20119^T^ (X83408) (98,26)	Lilihuapi Island	11.3	28.5	Shells and sponges	4–37	0–10	+	Bright cream	−	−	+
H-CG1	*Brachybacterium paraconglomeratum* JCM 17781^T^ (AB645761) (99.16)	Tambor Waterfall	6.1	30.5	Hard sediment	4–37	0–10	+	Bright yellow	+	+	+
H-BE7	*Brevibacterium oceani* BBH7^T^ (AM158906) (97.94)	Punta Llonco	25.1	29.5	Good water visibility	4–37	0–10	−	Bright orange	+	+	+
H-EH3	*Corynebacterium pilbarense* IMMIB WACC-658^T^ (FN295567) (98.10)	Loncochaigua River	0.25	5	Low tide	20–37	7	+	Bright cream	−	+	+
H-ED12	*Curtobacterium oceanosedimentum* ATCC 31317^T^ (GU269547) (99.02)	Punta Llonco	25.1	29.5	Good water visibility	20–45	0–10	−	Pale cream	−	+	+
H-KB5	*Kocuria polaris* CMS 76or^T^ (NR028924)(96.97)	Loncochaigua River	0.25	5	Low tide	4–37	0–10	+	Bright pink	−	+	+
H-KA4	*Dietzia natronolimnaea* DSM 444860^T^ (FJ468329) (99.06)	Tambor Waterfall	15.6	28.5	Sand, mussels and sea urchins	4–37	0–7	+	Intense orange	−	+	+
H-jCD9b	*Micrococcus luteus* NCTC 2665^T^ (CP001628) (99.15)	Punta Llonco	14.5	29	Shells, poor water visibility	20–37	0–7	−	Light yellow	−	−	−
H-CA8f	*Rhodococcus jianlingiae* djl-6-2^T^ (DQ185597) (98.84)	Lilihuapi Island	22.9	31	Shells and old nets	4–30	0	+	Light pink	−	+	+
H-KF8	*Streptomyces prasinus* NRRL B-2712^T^ (DQ026658) (99.92)	Punta Llonco	14.5	29	Shells, poor water visibility	4–37	0–7	+	White mycelium	−	+	+
H-CB3	*Streptomyces prasinus* NRRL B-2712^T^ (DQ026658) (99.86)	Tambor Waterfall	15.6	28.5	Sand, mussels and sea urchins	4–37	0–7	+	White mycelium	−	−	+

### Phylogenetic analysis

**Figure 4:**
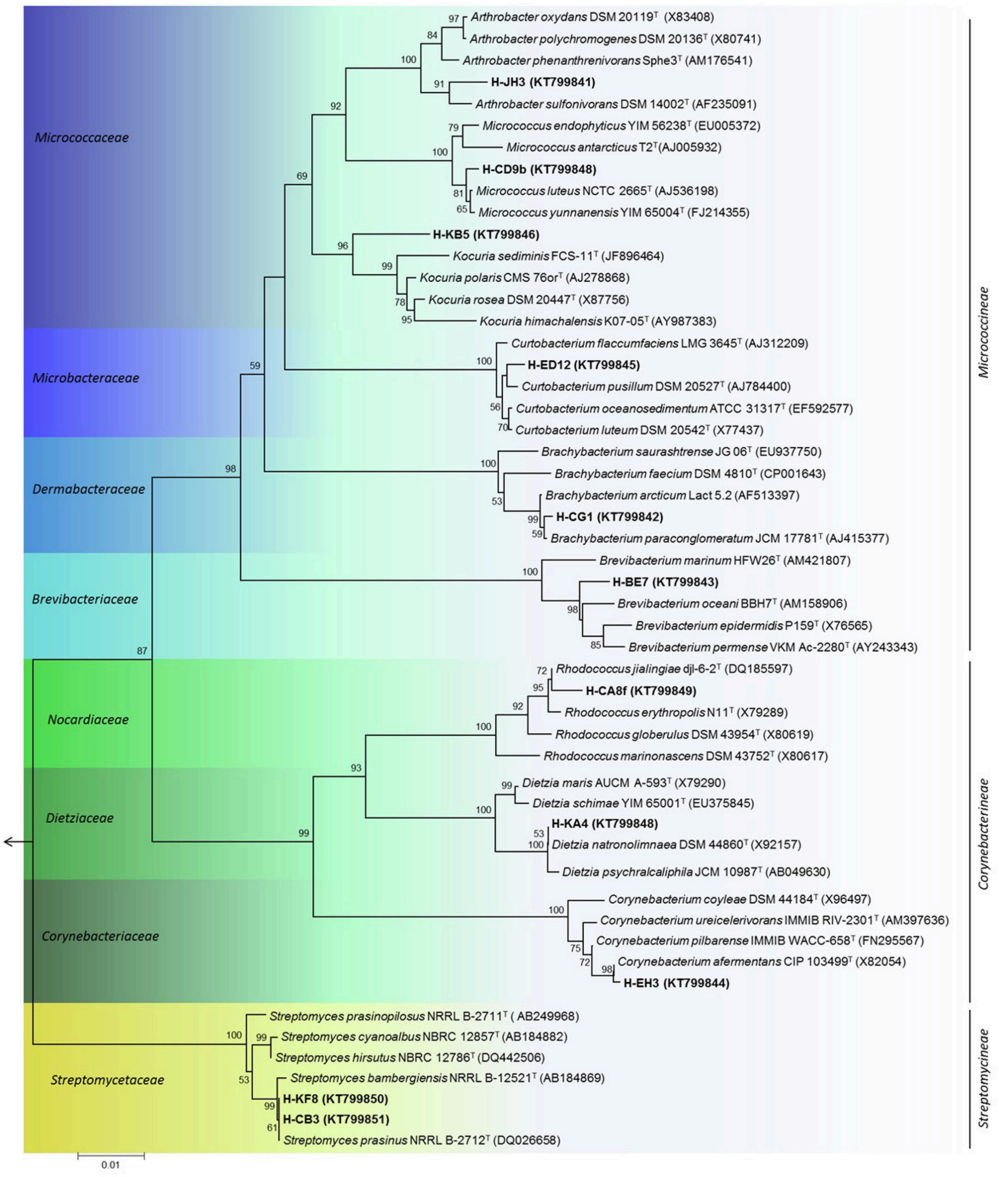
**Phylogenetic tree of representative actinobacterial strains isolated from the Comau fjord in Northern Patagonia, Chile**. Neighbour-joining tree of 16S rRNA gene showing the three suborders within the *phylum Actinobacteria*. Node numbers represent the percentage of bootstrap replicates (1000 resampling) which supported the proposed branching order shown at consistent nodes (values below 50% were not shown). Gene sequence positions 55–1410 were considered, according to the *Escherichia coli* K12 (AP012306) 16S rRNA gene sequence numbering. Arrow points to the outgroup *E. coli* K12. GenBank accession numbers of 16S rRNA sequences are given in parentheses. Scale bar corresponds to 0.01 substitutions per nucleotide positions.

For phylogenetic analysis, the 16S rRNA gene was sequenced for selected actinobacterial isolates, representatives of each genus retrieved in sediment samples from Comau fjord. A dendogram of the estimated phylogenetic relationships is presented in Figure 4 and the sequence similarities of selected actinobacterial strains to type strains of related species are given in Table 3. Four of the actinobacterial isolates are below the 98.7% sequence identity threshold and therefore may be potential candidates of new taxons. These isolates belong to *Arthrobacter* and *Kocuria* genera (*Micrococcaceae* family), *Brevibacterium* genus (*Brevibacteriaceae* family), and *Corynebacterium* genus (*Corynebacteriaceae* family) (Table 3). Interestingly, the psychrotolerant isolate *Kocuria* sp. H-KB5 has a 96.97% sequence identity with the type strain *K. polaris* CMS 76 or^T^, a strain isolated from an Antarctic cyanobacterial mat sample (Reddy et al., 2003). Moreover, strain H-KB5 forms a separate branch within the *Kocuria* group in the phylogenetic tree (Figure 4). This isolate will be further characterized in a polyphasic approach to determine its taxonomic position.

### Phenotypic characterization of isolated actinobacterial strains

**Figure 5:**
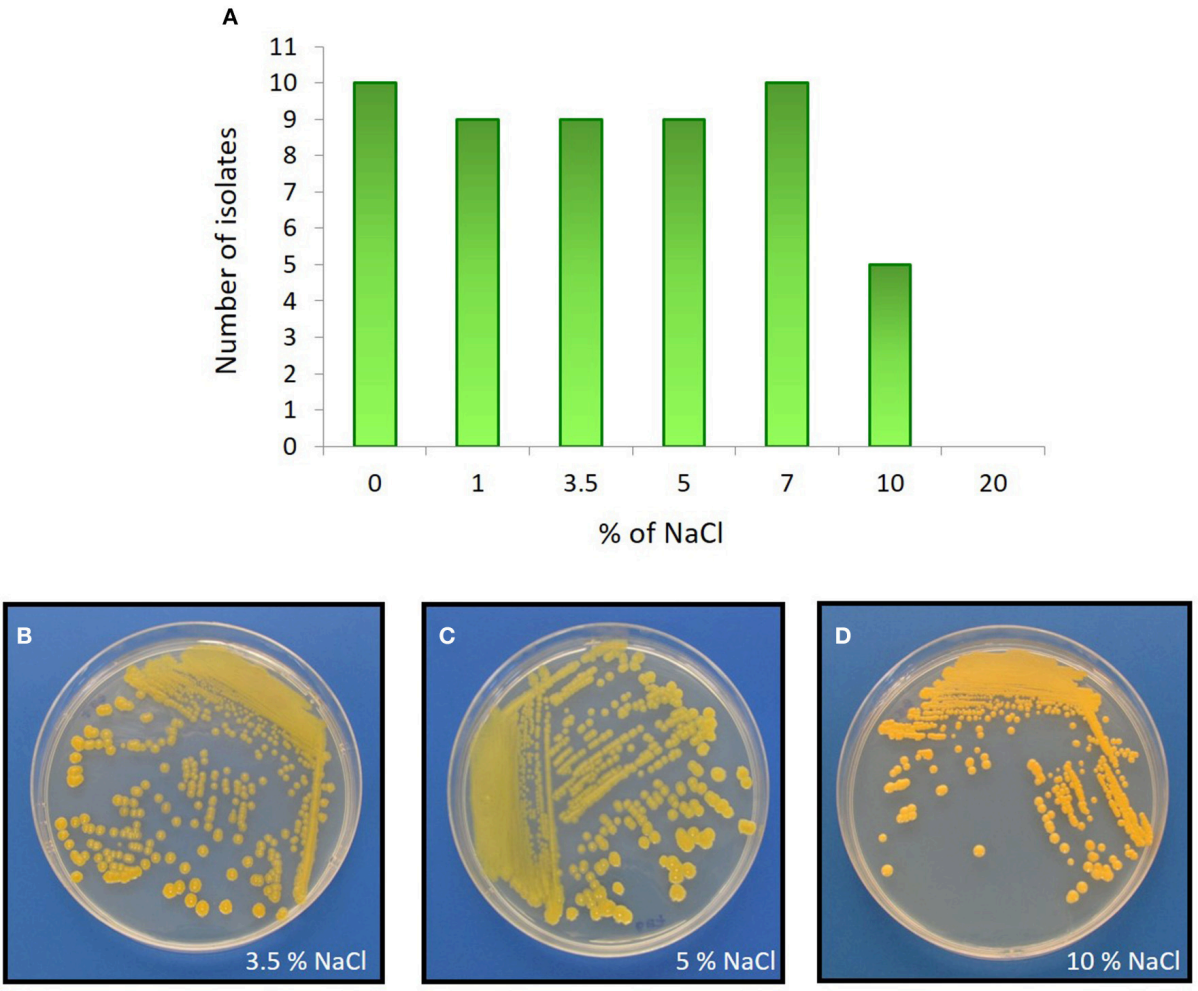
**NaCl effect on actinobacterial growth**. Upper panel: **(A)** Distribution of actinobacterial isolates and their ability to grow in LB medium with various percentages of NaCl. Bottom panel: As an example, the halophilic *Brevibacterium* sp. H-BE7 grown in LB medium containing: **(B)** 3.5%; **(C)** 5%; and **(D)** 10% NaCl concentrations.

The Comau fjord is characterized by defined zoning patterns of strong vertical and horizontal salinity gradients. The first 15 m underwater are influenced by waters of low salinity (~1.0%). Below this depth, a halocline is found that produces a constant water salinity of 3.2% (Castillo et al., 2012). In order to analyze how the salinity affects the growth of the actinobacterial isolates, NaCl tolerance was determined for each strain (Table 3). 82% of the representative isolates were able to grow in the presence of 1.0, 3.5, 5.0, and 7.0% (w/v) NaCl (Figure 5). 45% of the strains, belonging to *Arthrobacter, Brachybacterium, Brevibacterium, Curtobacterium*, and *Kocuria* genera, were able to grow in presence of 10% (w/v) NaCl (Table 3). None of the isolated actinobacteria was able to grow with 20% w/v NaCl.

To study adaptation to marine environments, actinobacterial strains were tested for ASW requirement. Most strains (73%), belonging to *Arthrobacter, Brachybacterium, Corynebacterium, Dietzia, Kocuria, Rhodococcus*, and *Streptomyces* genera were positively influenced by sea water as they required ASW for growth, suggesting marine adaptation. Interestingly, strain *Brevibacterium* sp. H-BE7, showed improved growth with both ASW and 3.5% NaCl, rather than with Milli-Q H_2_O and 0% NaCl, suggesting a specific salt requirement confirmed by its growth in 10% (w/v) NaCl (Figures 5B–D).

**Figure 6:**
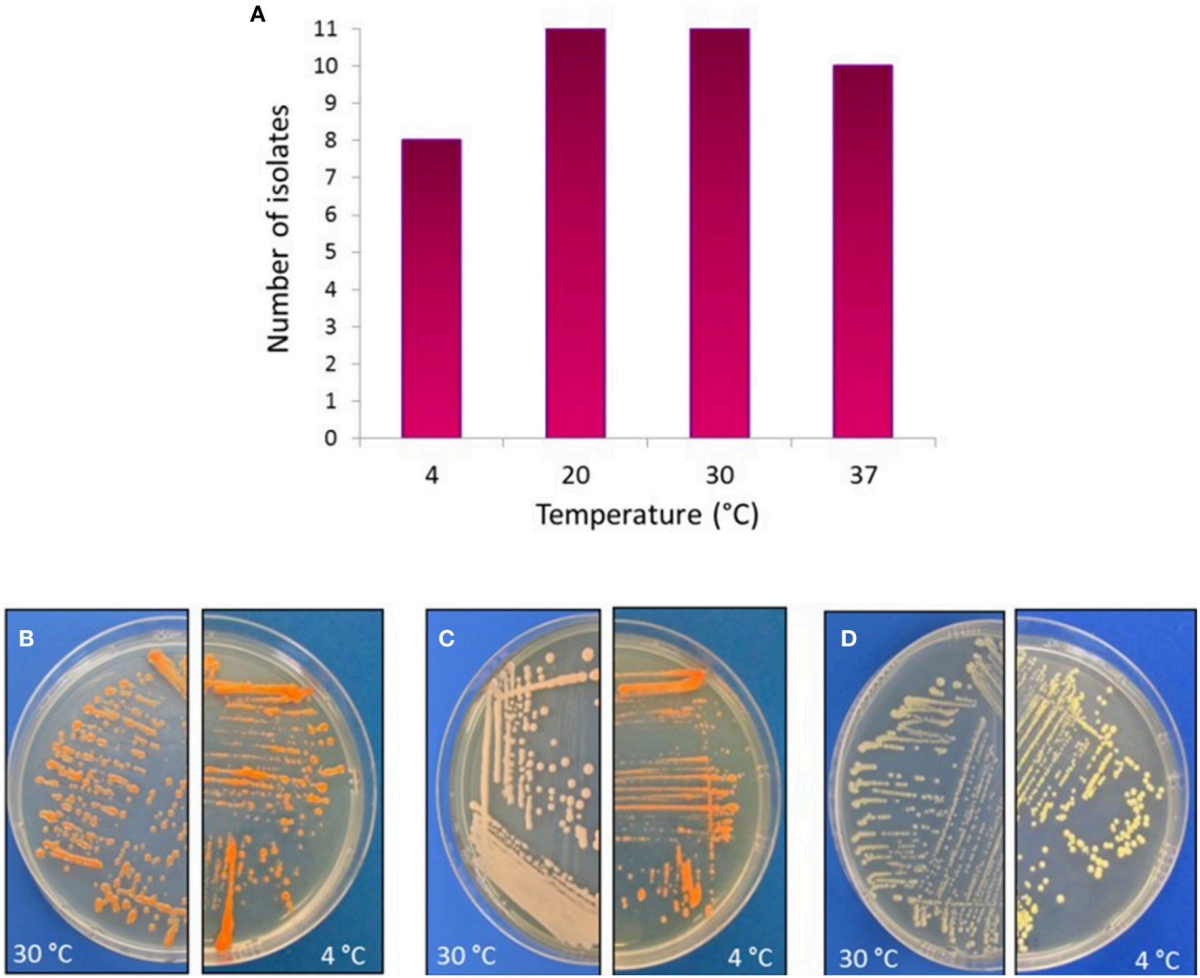
**Temperature effect on actinobacterial growth**. Upper panel: **(A)** Distribution of actinobacterial isolates and their ability to grow in different temperatures. Bottom panel: As an example, actinobacterial strains grown in TSA-ASW medium at either 30°C (left) or 4°C (right), showing differences in pigmentations. **(B)** *Dietzia* sp. H-KA4; **(C)** *Kocuria* sp. H-KB5; **(D)** *Brachybacterium* sp. H-CG1.

As the Comau fjord deep-waters reach temperatures below 10°C, actinobacterial strains were tested for growth at different temperatures. Notably, 73% of strains belonging to *Arthrobacter, Brachybacterium, Brevibacterium, Kocuria, Dietzia*, and *Rhodococcus*, and to a lesser extent, *Streptomyces*, were able to grow at 4°C (Figure 6). Moreover, pigmentation of the colonies was more intense after growth at 4°C, in comparison to 30°C (Figures 6B–D). Colony pigmentation of all representative actinobacteria was visualized macroscopically and detailed in Table 3.

### Resistance to model antibiotics

Antibiogram experiments demonstrated that all isolated actinobacterial strains are resistant to at least one of the antibiotics tested. Furthermore, these isolates showed resistance to several antibiotics of different classes. Interestingly, strains H-JH3, H-BE7, H-KA4, H-CD9, H-CG1, H-ED12, and H-CA8f showed resistances to ≥6 antibiotics, wherein resistance to tetracycline, ciprofloxacin and oxacyllin were observed for all the actinobacterial strains. Strain H-KA4 and H- ED12 showed resistance to all antibiotics tested, whereas strain H-BE7 was susceptible only for sulfonamides (Table 4).

**Table 4:** **Antibiotic resistance of selected actinobacterial strains**.

**Antibiotic**	**Class**	**Huinay Isolates**										
		**H-JH3**	**H-CG1**	**H-BE7**	**H-EH3**	**H-ED12**	**H-KB5**	**H-KA4**	**H-CD9**	**H-CA8f**	**H-KF8**	**H-CB3**
Penicillin (10 UOF)	β-Lactam	R	R	R	NG	R	R	R	R	R	R	S
Chloramphenicol (30 μg)	Other	S	S	R		R	S	R	R	R	R	R
Tetracycline (30 μg)	Poliketide	R	R	R		R	R	R	R	R	R	R
Oxacyllin (1 μg)	β-Lactam	R	R	R		R	R	R	R	R	R	R
Erythromycin (15 μg)	Macrolide	R	S	R		R	S	R	S	S	R	S
Clindamycin (2 μg)	Lincosamides	R	S	R		R	S	R	S	R	S	S
Sulfa-Trimethroprim (25 μg)	Sulfonamide	R	S	S		R	S	R	S	R	S	S
Ciprofloxacin (5 μg)	Fluoro-quinolone	R	R	R		R	R	R	R	R	R	R
Cefazolin (30 μg)	Cephalosporin (1st)	R	R	R		R	R	R	R	R	S	S
Gentamicin (10 μg)	Aminoglycoside	S	R	R		R	S	R	R	R	S	S

NG, No growth; R, Resistant; S, Susceptible.

## Discussion

Marine actinomycetes isolated from the National Marine Protected Area of Huinay at the Comau fjord in Northern Patagonia were studied, along with their physiological and taxonomic properties, and their potential to produce antimicrobial compounds. Patagonian fjords are largely unexplored, and may provide a rich source of microorganisms producing novel anti-infective compounds. This is the first bioprospection report of cultivable actinobacteria in this unique ecosystem, where 25 actinobacteria were isolated and characterized. Two studies report the isolation of marine actinobacteria from sediments of Chile's vast coast; one from Chiloé Island (Hong et al., 2010) and a recent study performed in Valparaíso Central Bay (Claverías et al., 2015). Only a metagenomic study has been carried out with a microbial mat located in the Comau fjord, revealing that 1% of community reads was represented by the *phylum Actinobacteria* (Ugalde et al., 2013).

In this study, a lower abundance of actinobacteria associated to marine sediments was observed compared to Valparaíso Bay where actinobacterial strains belonging to 18 genera were isolated, using the same cultivating conditions (Claverías et al., 2015). Although, members of the *Rhodococcus* and *Dietzia* genera were successfully isolated from the Comau fjord, they were less represented (8%) than in Valparaiso Bay (33%). The lower actinobacterial abundance in Comau fjord could be due to the lower content of organic matter in this microhabitat that can range between 0.5 and 3.4% of organic carbon content for Northern Chilean Patagonian fjords (Sepúlveda et al., 2011). Gram-positive bacteria are more commonly observed in organic rich habitats (Fenical, 1993). Water samples from Valparaíso Bay are influenced by contamination with polycyclic aromatic hydrocarbons as well as with heavy metals (Campos et al., 1987; Palma-Fleming et al., 2008; Fuentes et al., 2015). It can also be influenced by hydrographic features such as seasonal upwelling which can supply nutrients to shallow waters (Capone and Hutchins, 2013). In contrast, the Comau fjord has a high precipitation rate that provides a fresh water input (Silva, 2006) which can affect microorganisms in marine sediments. The four sites from Comau fjord have minimal anthropogenic intervention, thereof changes in microbial communities are given almost exclusively by natural processes.

Despite the fact that a relatively low number of actinobacterial strains were retrieved from Comau fjord, a rather high cultivable biodiversity (10 genera) was observed using 5 isolation media. In comparison, the actinobacteria isolated using 11 selective media from the Trondheim fjord (Norway) belonged to 12 genera (Bredholdt et al., 2007). Also, in a culture-dependent study using sediments collected near Chiloé Island, Chile, five genera were retrieved using 7 media, being dominant the *Micromonospora* genus (Hong et al., 2010). Although, no *Micromonospora* members have been isolated in this work, this could be due to the different isolation media used. In this report, 24% of isolates were obtained from NaST21Cx medium, which is derived from ST21Cx medium by elimination of yeast extract and replacement of artificial sea water (Magarvey et al., 2004). It has been reported that media composed of relatively simple nutrients yielded more cultured actinobacteria in diverse environments (Zhang et al., 2006; Gontang et al., 2007; Qin et al., 2012). This is consistent with the negligible amount of nutrients that are actually available for marine actinobacteria within hostile ocean ecosystems (Das et al., 2006). This is the case for our study since more isolates were obtained with media containing low nutrients or complex carbon sources rather than common media constituents such as peptone and simple sugars, which are proposed to be unrealistic marine nutrients (Kurtböke et al., 2015). In this study, the major abundance of actinobacteria was found in deeper samples, which is in accordance with that observed in the Trondheim fjord (Hakvåg et al., 2008). Moreover, an elevated number (73%) of isolates showed an ASW requirement for growth. Evidence of isolation of seawater-dependent actinobacteria from marine sediments has been reported (Mincer et al., 2002; Maldonado et al., 2005). The fact that growth of some isolates is positively influenced by sea water can be an indicator that suggests they might be well adapted to the marine environments (Bredholdt et al., 2007; Penn and Jensen, 2012; Yuan et al., 2014). Nevertheless, since isolates obtained from Comau fjord can also grow without NaCl, they represent novel moderate halotolerant features in actinobacteria from this pristine sampling zone. This is consistent with the fact that these isolates have to overcome the dynamics of strong salinity gradients observed within the Comau fjord.

Reports of marine actinomycetes as a source of novel secondary bioactive metabolites have been extensively recognized (Haefner, 2003; Knight et al., 2003; Fiedler et al., 2005; Fenical and Jensen, 2006; Zhang et al., 2006; Gulder and Moore, 2010; Kurtböke et al., 2015). Two screenings for antimicrobial activities were pursued in this report, and notably, inhibition of the growth of at least one of the model bacteria was observed. It is noteworthy to highlight that antimicrobial activities from non-mycelial strain (e.g., *Rhodococcus* sp. H-CA8f) outcompete the activities of mycelial-type strains. To our knowledge, this is the first report of strong antibacterial activities associated to a *Rhodococcus* isolated from marine sediments. The *Rhodococcus* strain isolated in this study has a strong activity (>50% growth inhibition) against *E. coli, S. enterica, P. aeruginosa*, and *L. monocytogenes*; whereas a *Rhodococcus* strain isolated from Valparaíso Bay sediments (Claverías et al., 2015) had only a modest activity against *E. coli*. Antimicrobial activity from marine-derived isolates, but not necessarily from sediments, includes a *Rhodococcus* isolated from South China Sea corals that presented activity against *B. subtilis, B. thuringiensis*, and *E. coli* (Zhang et al., 2013), whereas *Rhodococcus* strains isolated from corals of the Arabian Gulf showed activity against *S. aureus* (Mahmoud and Kalendar, 2016). In this study, antimicrobial activity of *Arthrobacter* sp. H-JH3 against *S. enterica* and *E. coli* is highlighted by its novelty. In this line, there are reports about antarctic *Arthrobacter* strains isolated from sponges that were able to inhibit the growth of *Burkholderia cepacia* complex by the production of volatile organic compounds (Fondi et al., 2012; Orlandini et al., 2014). Also, antimicrobial activity against *Vibrio anguillarum* and *S. aureus* was detected from samples collected from the Arctic Ocean (Wietz et al., 2012). Interestingly, this is the first report indicating growth inhibition of Gram-negative strains by a *Brevibacterium* isolate. Only a bacteriocin able to inhibit *L. monocytogenes*, but inactive against Gram-negative was reported for this genus (Motta and Brandelli, 2002). In contrast, antimicrobial activity against *S. enterica* was observed in crude extracts, suggesting a different mode of action.

It has been reported that most natural products with interesting biological activities are synthesized by PKS (type I or type II), NRPS, and even PKS-NRPS hybrid pathways (Fischbach and Walsh, 2006). Some pharmacologically commercial examples include the polyketide antibiotic erythromycin (Staunton and Wilkinson, 1997) and the non-ribosomal peptide antibiotic of the cephalosporin family (Aharonowitz and Cohen, 1992). In this report, a PCR-based screening was pursued for the detection of these biosynthetic genes in actinobacterial isolates, in order to explore the potential to produce secondary metabolites with biotechnological applications. Notably, 91% of the isolates tested showed the presence of at least one of the three biosynthetic genes, which confirms that these metabolic pathways are widely distributed among this *phylum* (Donadio et al., 2007). As molecular methods for analyzing these genes are useful for screening of isolates for prediction of potential bioactive molecule production (Hodges et al., 2012), future efforts will be focused in sequencing these biosynthetic genes, to gain knowledge of the novelty of the bioproducts in which they are involved in.

The marine habitat sampled in the Northern Patagonia of Chile was a promising scenario to search for novel actinobacterial strains. In this study, four putative new species are proposed: *Arthrobacter* sp. H-JH3, *Brevibacterium* sp. H-BE7, *Corynebacterium* sp. H-EH3 and *Kocuria* sp. H-KB5, based on numerical thresholds related to 16S rRNA gene sequences (Rosselló-Móra and Amann, 2015). In addition, representatives of *Micrococcineae, Corynebacterineae*, and *Streptomycineae* suborders were isolated. Interestingly, actinobacterial isolates showed sequence similarity with strains reported from colder habitats. 73% of the isolates belonging to *Arthrobacter, Brachybacterium, Brevibacterium, Kocuria, Dietzia, Rhodococcus*, and *Streptomyces* genera were able to grow at 4°C, suggesting a psychrotolerant adaptation which is in accordance with the water body temperature range of the Comau fjord (Lagger et al., 2009; Sobarzo, 2009), sustaining a thermohaline circulation (Bustamante, 2009). A difference in colony pigmentation was observed at low temperatures. Pigments can be enhanced under specific conditions such as climate stress, since they are part of the non-enzymatic antioxidant mechanisms in cell defense to prevent oxidative damage (Correa-Llantén et al., 2012). Another role of pigments in response to cold is to decrease the membrane fluidity to counterbalance the effects of fatty acids in Antarctic bacteria (Chattopadhyay, 2006). Pigments can also contribute to antibacterial activity, positioning them as interesting biotechnological candidates for food, cosmetic and textile industries (Rashid et al., 2014; Leiva et al., 2015).

Comparison with 16S ribosomal RNA sequences Bacteria and Archaea NCBI database, reveals only two closest type strains of marine origin: *Brevibacterium oceani* BBH7^T^ isolated from deep sea sediment of the Indian Ocean (Bhadra et al., 2008) and *Curtobacterium oceanosedimentum* ATCC 31317^T^ isolated from Irish sea marine sediments (Kim et al., 2009). In contrast, when sequences are compared with NCBI nucleotide collection database, actinobacterial isolates showed more similarity with polar marine isolates. This is the case for the psychrotolerant *Arthrobacter* sp. H-JH3, which showed a 98.82% identity with *A. scleromae* Asd M4-11 (Vardhan Reddy et al., 2009), a bacterium isolated from a melt water stream of an Arctic glacier. The psychrotolerant *Brachybacterium* sp. H-CG1 showed a high similarity (99.16%) with *B. articum* Lact 5.2 (Acc. Number: AF434185, unpublished), a bacterium isolated from a sea-ice sample from the permanently cold fjord of Wijde fjord, Spitzbergen, in the Arctic Ocean. Another interesting relation is given for strain H-CD9b from the genus *Micrococcus*, which has a 99.15% of sequence identity with the type strain *M. luteus* NCTC 2665^*T*^ (Rokem et al., 2011) that is a soil metal resistant bacterium, and a slightly more sequence identity (99.43%) with *Micrococcus* sp. strain MOLA4 (Acc. Number: CP001628, unpublished) a bacterium isolated from sea water of North Western Mediterranean Sea. Also, strain H-CA8f, showed a higher sequence similarity (98.91%) to *Rhodococcus* sp. TMT4-41 isolated from a glacier in China (Acc. Number: JX949806, unpublished) than to its closest type strain *R. jialingiae* djl-6-2^T^ (Wang et al., 2010).

Antibiogram experiments demonstrated that, in general, actinobacterial strains showed resistance. Interestingly, *Curtobacterium* sp. H-ED12, *Dietzia* sp. H-KA4 and *Brevibacterium* sp. H-BE7 showed resistance to almost all antibiotics tested, possibly due to the presence of multiple biosynthetic clusters, involving different classes of antibiotic compounds. Strains H-BE7 and H-ED12 inhibited both Gram-positive and Gram-negative model bacteria, suggesting different modes of action of the antibacterial molecules produced by this strain. Thus, it seems plausible that biosynthetic pathways involving metabolites of similar nature could be present in these isolates. A typical cluster of secondary metabolism includes genes for multi-domains enzymes that carry out the synthesis of different bioactive metabolites and when this metabolite has an antimicrobial activity, it is coupled to its corresponding resistance gene (Zotchev, 2014).

To our knowledge, this is the first report of the isolation and ecophysiological characterization of actinobacteria from sediments of a Patagonian fjord. This single survey uncovered a broad cultivable diversity which provides the basis for the bioprospection of bioactive compounds. The isolation of novel actinobacterial species and the evidence that most of our isolates produced antibiotic activities supports our approach.

## Author contributions

AU conceived and designed the experiments, performed the experiments, analyzed the data, prepared the manuscript. FB designed the experiments. FC performed the experiments. MG performed the sampling and experiments. EM performed the sampling and edited the manuscript. MS performed the sampling, prepared and edited the manuscript. BC performed the sampling, conceived and designed the experiments, analyzed the data, prepared and edited the manuscript.

### Conflict of interest statement

The authors declare that the research was conducted in the absence of any commercial or financial relationships that could be construed as a potential conflict of interest.
